# Spatial-temporal differentiation pattern and influencing factors of land economic density at the township scale in Zhejiang Province

**DOI:** 10.1371/journal.pone.0304327

**Published:** 2024-05-31

**Authors:** Fangfang Ma, Yiping Hu, Zhiwei Ding

**Affiliations:** 1 The College of Geography and Environmental Science, Henan University, Kaifeng, Henan, China; 2 Key Research Institute of Yellow River Civilization and Sustainable Development Collaborative Innovation Center on Yellow River Civilization, Kaifeng, Henan, China; 3 National Demonstration Center for Environment and Planning, Henan University, Kaifeng, Henan, China; 4 Zhejiang Tourism Investment Group, Hangzhou, Zhejiang, China; Northeastern University (Shenyang China), CHINA

## Abstract

Based on the land economic density of 892 town units, the spatial pattern of the land economic density in Zhejiang Province is analyzed using the coefficient of variation, spatial classification, and spatial correlation methods, and the influencing factors are analyzed using a spatial regression model. The results are as follows: (1) The coefficients of variation were 2.6 and 3.1 in 2014 and 2019, respectively, indicating that the degree of imbalance of the town’s industrial economy at the county level increased. (2) The distribution of the high-level agglomeration areas was characterized by one core area and two sub-core areas. The main core area was located at the junction of Hangzhou City, Shaoxing City, and Jiaxing City, and the two sub-core areas were located in Yuyao City and the main urban area of Ningbo City. In addition, several small-scale agglomeration areas composed of medium and high-level units were distributed in Wenzhou City. (3) The high-value agglomeration and low-value agglomeration distribution in the spatial correlation patterns was identified using the spatial auto-correlation method. The hot spots and sub-hot spots were distributed in Northern Zhejiang, and the cold spots formed a large-scale agglomeration in Quzhou City, Lishui City, Taizhou City, and several other cities in Southern Zhejiang. (4) Compared with the county scale, the spatial scope of the high-level areas in Northern Zhejiang shrunk significantly at the township scale, and the high-level agglomeration areas along the southeast coast changed into a cluster of several townships. (5) According to the geographically weighted regression (GWR) model, the importance of influencing factors is as follows: population density > regional area > industrial output value per capita > total population > proportion of secondary and tertiary personnel > total employees.

## Introduction

From the microscopic perspective of regional economics, the township economy is of great significance for supporting rural revitalization, agricultural modernization, and urban-rural integrated development. In recent years, the township economic force has been continuously improved and the urban-rural income gap has been improved, which has also promoted urban-rural integrated development with the strategic implementation of new urbanization and rural revitalization in China. As an important natural resource and a space for economic-social development, town land plays an important role in supporting resource development activities and improving economic output benefits. In the central business districts (CBDs) of metropolitan areas, because of the high cost of investments, land economic density is an indicator of the intensity of economic activities, which reflects the actual investment efficiency and potential output value. However, owing to the low intensity of human activities and the distance from the market, investors are reluctant to undertake land-related economic activities in some underdeveloped areas, especially in towns with poor geographical conditions. To promote the high-quality development of land resources, the state regularly conducts land economic evaluations and conducts land use planning within a certain period to ensure improvement of the economic quality and optimization of the industrial structure. Therefore, previous studies have paid attention to the government’s land strategies and used the land economic density to reflect the input-output benefit of economic activities. The current research on land economic density predominantly focuses on four key aspects. Firstly, the aspect of measuring the level of land economic density. Since the land economic output is vary in different regions, the indicators that reflect the land economic density include not only the gross economic output such as the GDP but also several sector and industry indicators such as the industrial output value [1, 2], fiscal revenue [3, 4], investment in fixed assets [5], and some non-agricultural activity values [6]. The built-up area serves as the primary spatial carrier of urban economic activities, therefore, the economic value added per unit of built-up area is a common indicator for measuring land economic density [7]. Among them, urban construction land is the most developed and fully utilized land type among all types of land, and the research on the economic density of urban construction land has a strong typicality [8]. Additionally, measuring the economic density of urban and rural construction land can highlight the contribution of rural construction land to the regional economy, which can more comprehensively reflect the economic benefits of land use [9]. Secondly, with regard to the models used for the land economic density, the main models used are urban economic density and regional economic density models. Urban economic density models include population models, urban spatial structure density models, and gravity models [10, 11]. Regional economic density models include population density models, such as empirical models (e.g., the Parr model) and economic models (e.g., the Beckman-Weibull model) [12]. From the perspective of land economic density and territorial spatial planning, most studies have analyzed the relationship between the land development mode and the urban spatial layout based on the regional location, industrial structure, and input-output benefits of different types of land, which provides theoretical support for urban-rural modernization construction [13, 14]. The third dimension involves the spatiotemporal evolution of land economic density. Researchers commonly utilize models such as the Theil index, coefficient of variation, Markov chain, kernel density estimation, spatial autocorrelation, among others [15–17], to investigate the spatial differentiation [18], dynamic evolution [19], and spatiotemporal changes [20] in land economic density at different scales. In terms of spatial characterization, some researchers have studied the interaction mechanisms between the land economic density and factors such as corporate activities, industrial agglomeration, real estate investments, industrial diversification, and regional innovation. From the perspective of the temporal characteristics, most researchers have studied the dynamic changes within a certain time period in different countries and provinces based on the overall land economic density and urban land economic density and have analyzed the phase characteristics and evolution trends to better regulate economic activities in the future [21–24]. Fourth, in terms of research on influencing factors, most researchers have investigated the relationship between the land economic density and urban modernization construction using factors such as the population scale, economic vitality, industrial positioning, location conditions, and urbanization processes via empirical analysis. Several researchers have conducted qualitative analysis from the aspects of the leading industry, enterprise strategy, and government policies [25, 26]. Additionally, Wu et al., approaching from the perspective of resource endowment, found that the level of land economic density varies in different regions due to differentiated impacts of natural resource endowment [27]. Cao et al., included geographic location in their analysis and found that port locations such as coastal waterways, inland waterways and airways have different effects on land economic density [28]. In summary, although area-specific research has been continuously strengthened, few studies have been conducted on specific provinces, cities, and counties at micro-scales such as the township and village scales, and the depth of the analysis of the influencing factors also needs to be strengthened. Therefore, exploring the spatial differentiation of the land economic density in specific regions at the township and village scales and determining its influencing factors and proposing targeted solutions for specific problems can effectively provide support for regional spatial planning and optimization of the spatial layout.

## Materials and methods

### Data sources

**Fig 1:**
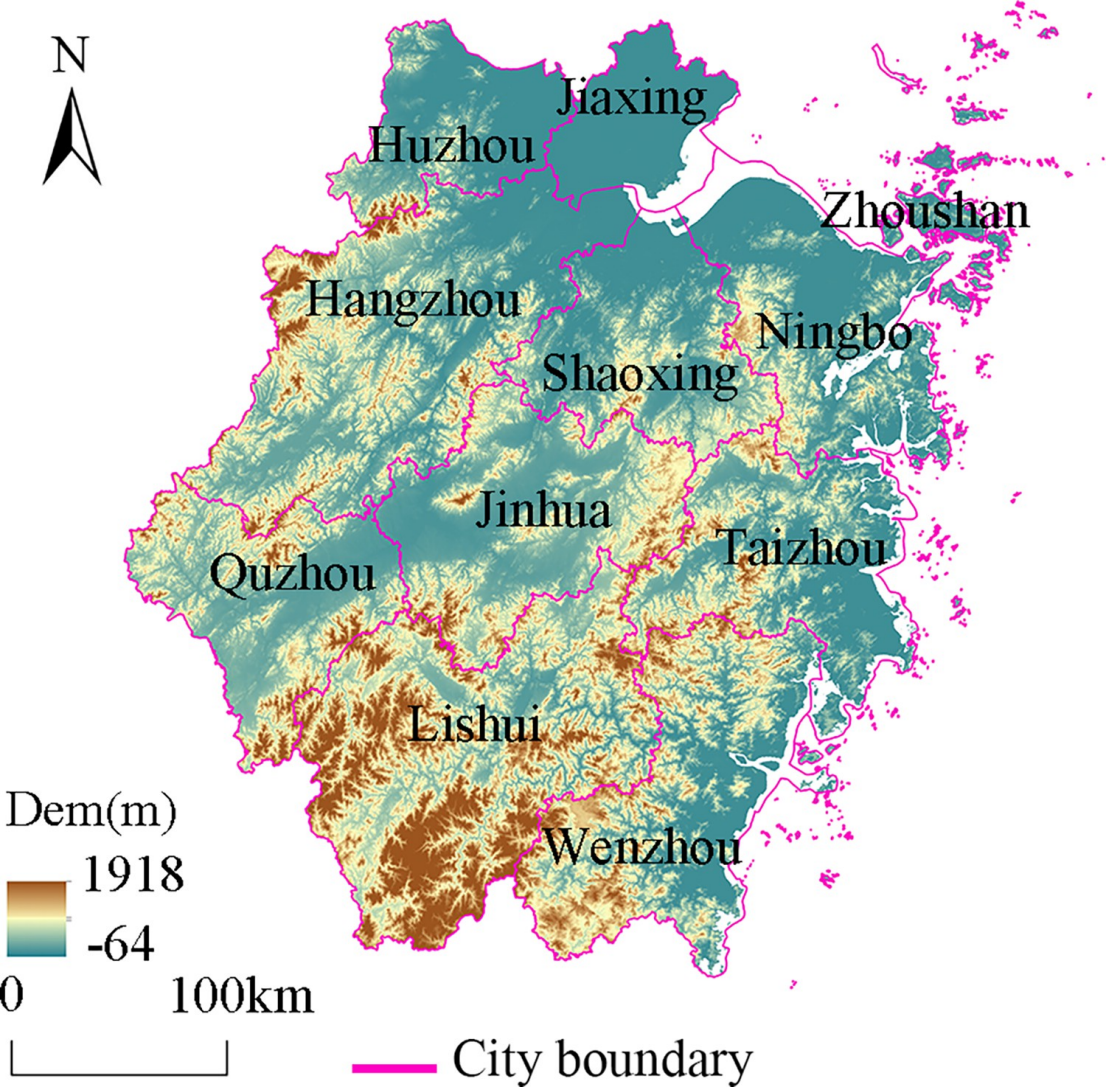
Elevation map of Zhejiang Province.

Zhejiang Province is a core region in national strategic planning initiatives such as the Silk Road Economic Belt, the coordinated development of the Beijing-Tianjin-Hebei region, and the Yangtze River Economic Belt, consistently maintaining a prominent position in China’s GDP rankings. The province’s development is primarily reliant on its advantageous economic geographical location and the thriving private economy at the township level. However, variations in natural conditions, infrastructure, and policy support within the region have led to disparities in the economic development foundations at the township level (Fig 1). Zhejiang Province contains 1,346 townships (including urban street management areas) in 11 cities. It should be noted that although the urban street management areas are of the township scale, there are great differences in their administrative modes, economic structures, and organizational frameworks. Therefore, the urban street management areas were excluded, and we ultimately identified 892 research units. The data were obtained from the county yearbooks and China’s Counties Statistical Yearbook (Township Volume), government work reports, and official websites.

Research methods

#### Land economic density

The land economic density is measured by the total industrial output per unit area of the administrative region and is calculated by the formula:

L=T/S (1)

where *L* is the land economic density, *T* is the total industrial output value, and *S* is the area of the administrative region.

#### Coefficient of variation

The coefficient of variation *CV* can effectively reflect the state of the internal economic imbalance [29]. The *CV* is calculated as follows:

$$C_{V}=\frac{s}{\bar{x}}$$ (2)

where *CV* is the coefficient of variation of a study unit; and S and $\bar{x}$ are the standard deviation and mean of the study unit, respectively.

#### Spatial interpolation analysis

Kriging interpolation, also known as spatial local interpolation, is a method for unbiased optimal estimation of a variable in a study area based on the theory of variational functions and structural analysis [30].

#### Spatial autocorrelation analysis

As an ESDA method, the cold–hot spot analysis method is used to identify spatial clusters with statistically significant high values (hot spots) and low values (cold spots). The specific formula is as follows:

$$G_{i}(d)=\frac{\sum_{j=1}^{n}w_{ij}(d)x_{j}}{\sum_{j=1}^{n}x_{j}}(i\neq j),\;Z(G_{i})=\frac{G_{i}-E(G_{i})}{\sqrt{VarG_{i}}}$$ (3)

where the positive and negative values of Z(*G*_*i*_) indicate that the values around the cluster areas are hot spots and cold spots, respectively; *W*_*ij*_ is the spatial weight of the research area, and the spatial adjacent and non-adjacent states are 1 and 0. E(*G*_*i*_) and Var(*G*_*i*_) are the mathematical expectation and variance of *G*_*i*_, respectively.

## Results and analysis

### Degree of intra-regional differences

**Fig 2:**
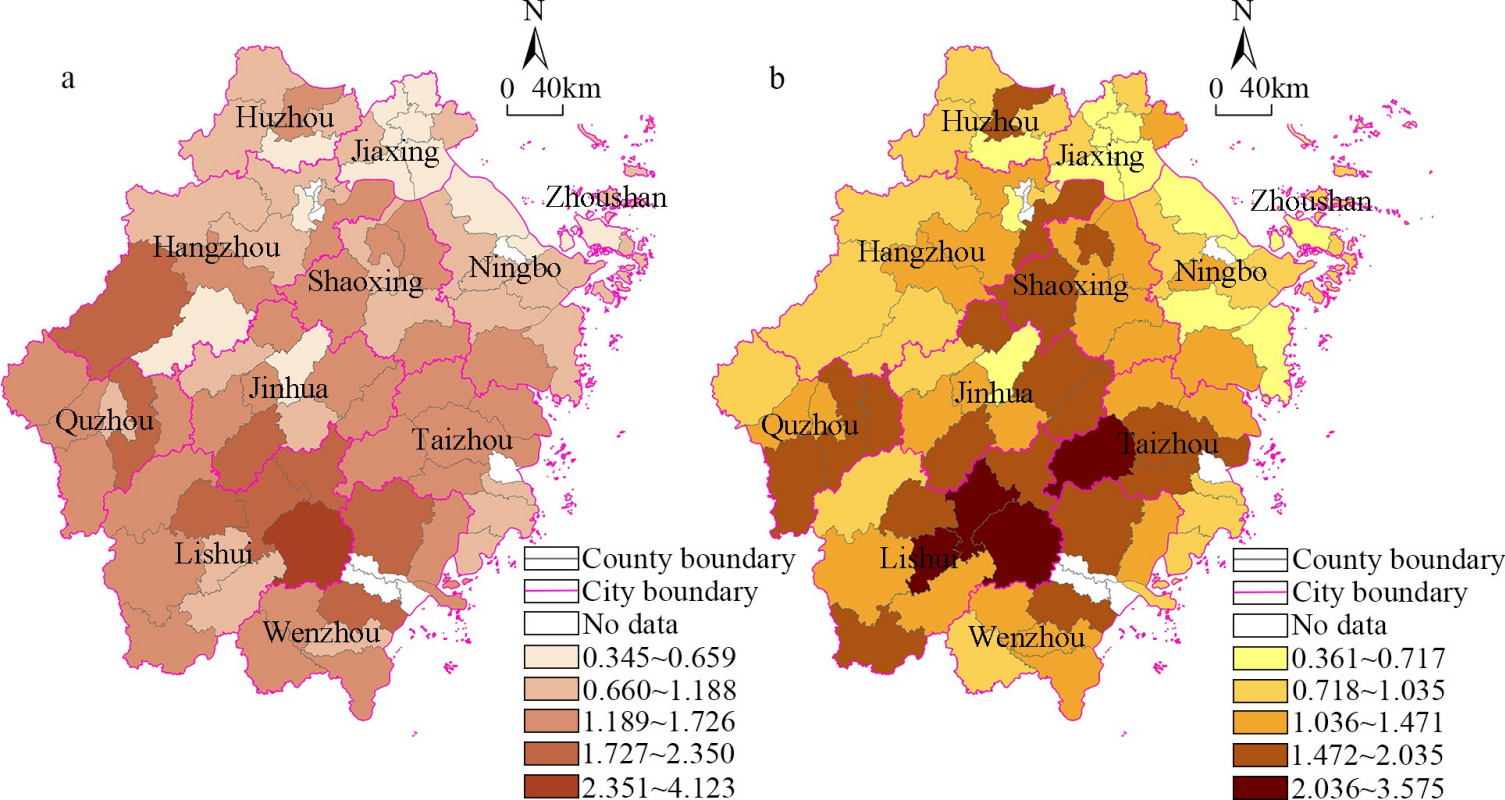
Spatial variations in the land economic density in the townships in Zhejiang Province from 2014 to 2019. (a)Year 2014(b)Year 2019.

Analyzing the spatial distribution of the coefficient of variation (*CV*) in 2014 (Fig 2), counties with higher levels of land economic density equilibrium were primarily located in cities such as Jiaxing, Ningbo, Hangzhou, and others. Counties with a generally balanced land economic density were mainly found in the central areas, including Jinhua, Taizhou, and Quzhou. Counties with low levels of land economic density equilibrium were concentrated mainly in Lishui City. A comparison with 2019 reveals an increase in counties with lower land economic density equilibrium levels, mainly distributed in cities such as Lishui, Wenzhou, Taizhou, Jinhua, among others. Counties with higher levels of land economic density equilibrium continued to be concentrated in cities like Jiaxing, Ningbo, and Hangzhou. As a whole, there is little overall change in the spatial pattern between 2014 and 2019, therefore, in this study, 2019 was taken as an example to conduct in-depth research on the spatial differences. The high variance areas with *CV* values of 2.036–3.575 were distributed in the northeastern part of Lishui City, and the areas with medium-high *CV* values of 1.472–2.035 were mainly distributed around the high-level units, forming a large-scale agglomeration with high variance areas in central-southern Zhejiang. In practical terms, these regional county economies are constrained by the local industrial structure, lacking diversified industrial support. Simultaneously, the lag in infrastructure and industrial chain development has resulted in a relatively lower level of economic development in the townships of these areas, forming an internal imbalanced economic pattern. The medium variance areas with *CV* value of 1.036–1.471 were distributed in central-northern Zhejiang and its southern outskirts. The low variance areas with *CV* values of less than 1.036 were mainly distributed in northern Zhejiang, which is similar to the distribution pattern of the high-level economic areas at the county scale. It was found that these regions not only had small urban-rural income gaps, but that the township economies were always in a state of high growth. Thus, the spatial distribution of the township economies was in a state of high-level equilibrium, and the internal degree of variance was small. In general, driven by the high level of the county economy, the strengths of the towns in northern Zhejiang differed little from each other, resulting in a highly balanced state.

### Spatial agglomeration pattern

**Fig 3:**
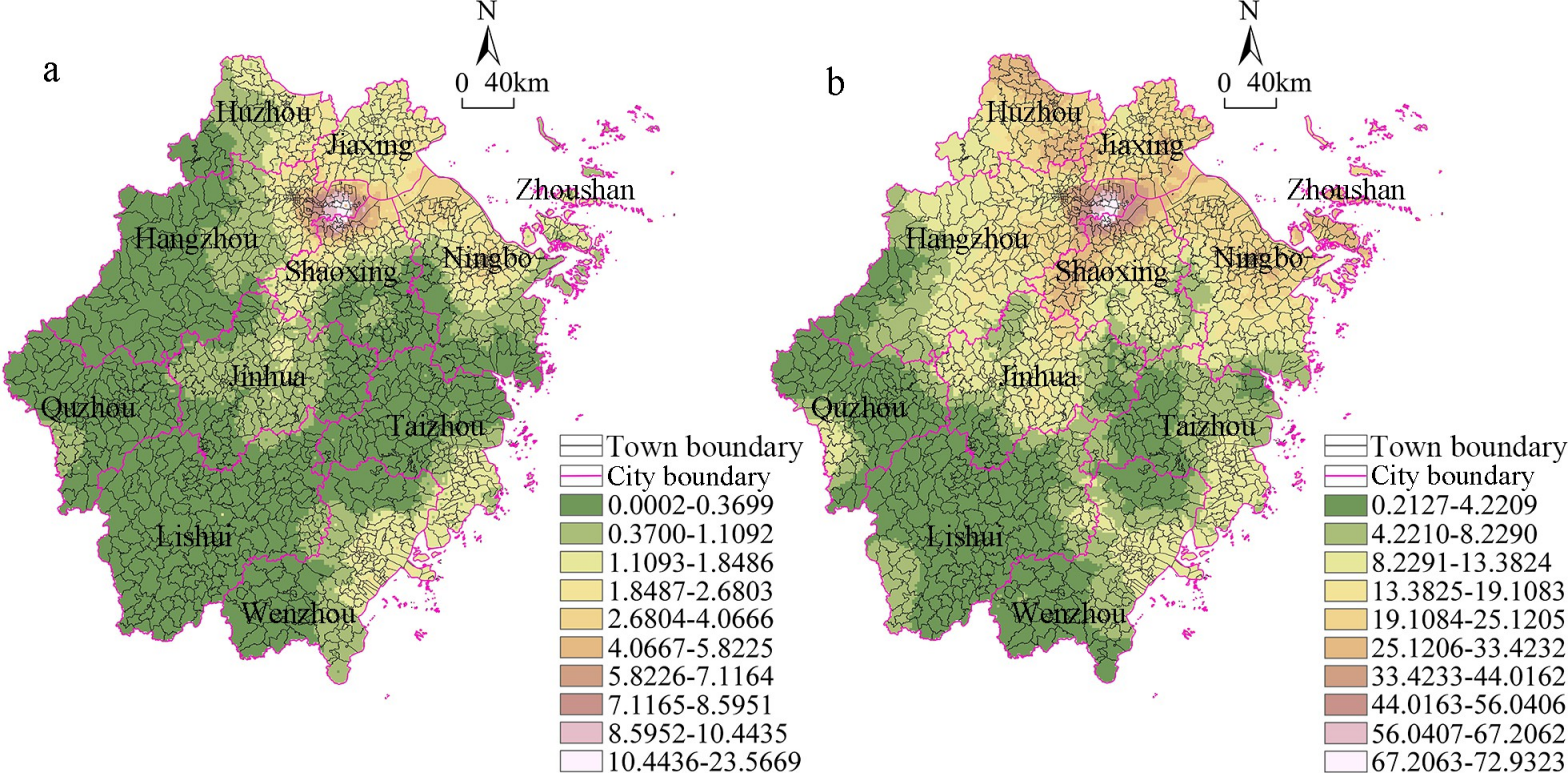
Spatial differences in the land economic density in the townships in Zhejiang Province from 2014 to 2019. (a)Year 2014 (b)Year 2019.

The kriging interpolation method as used to visualize the land economic density at the township scale, and the results are shown in Fig 3. In 2014, the high-value agglomeration pattern was characterized by one main core area and two sub-core areas. The main core area was located in the northern border area between Hangzhou City and Shaoxing City, mainly including the Xiaoshan District, Binjiang District, and Shangcheng District. These areas had superior geographical locations and convenient transportation conditions, which encouraged many foreign-funded enterprises to invest and build factories, resulting in the integrated development of industry and service industries. The two sub-core areas were located in Yuyao City and the main urban area of Ningbo City, mainly including the Yinzhou District, Jiangbei District, Haishu District, and Beilun District. At present, these areas are important ports in China, they have the combined advantages of integrated logistics, e-commerce, and other service industries and cooperate closely with industrial enterprises to form a coupled population-land-industry model. Under the multiple functions of industrial upgrading and urban construction, these areas continuously improved the comprehensive utilization efficiency of the land. In addition, the main core areas extended around and connected with the two sub-core areas, forming a high-level agglomeration area in northern Zhejiang. Hangzhou, a rapidly developing area in northern Zhejiang, was taken as an example. It contained a consolidated core area, and it also led and drove industrial economic development of the surrounding villages and towns. It should be noted that the southeastern part of Wenzhou also exhibited a strip-like high-value distribution pattern. This was mainly due to the light industrial commodities and family workshops in the Wenzhou model, which resulted in its township economy having a unique development path. Therefore, the overall township economic pattern of Wenzhou City was mainly dominated by a medium and high-value distribution. Low-level areas were numerous and were widely distributed in southern Zhejiang, including in most areas of Lishui, Quzhou, Taizhou, and other cities. From a geographic perspective, these areas were mainly mountainous and hilly, which is not conducive to the traffic layout and the rational development of resources and limits the development of the regional industrial economy. Compared with 2014, in 2019 the number of high-level agglomeration areas remained unchanged, but the values in the main core area decreased. With the optimization and upgrading of industrial structure, the core area is gradually transitioning towards high-tech industries and the service sector, reducing its dependence on traditional manufacturing. Simultaneously, in response to changes in the market environment and shifts in consumer demand, some traditional industrial products are experiencing a decrease in market demand. In light of these challenges, industrial enterprises in the core area have undertaken adjustments to their production structures, seeking new avenues for growth. The number of low-level agglomeration areas decreased significantly, but the values increased. With the sustained development of Zhejiang Province’s economy, the costs of land, labor, and environmental protection requirements in the core area have been steadily increasing. This trend has prompted some manufacturing and industrial enterprises to relocate to lower-cost, less developed areas, thereby enhancing the economic density of these regions. Concurrently, the government has extended support to these lower-level areas through infrastructure development, tax incentives, technological assistance, and other measures, stimulating industrial growth and economic expansion in these regions.

### Spatial correlation analysis

**Fig 4:**
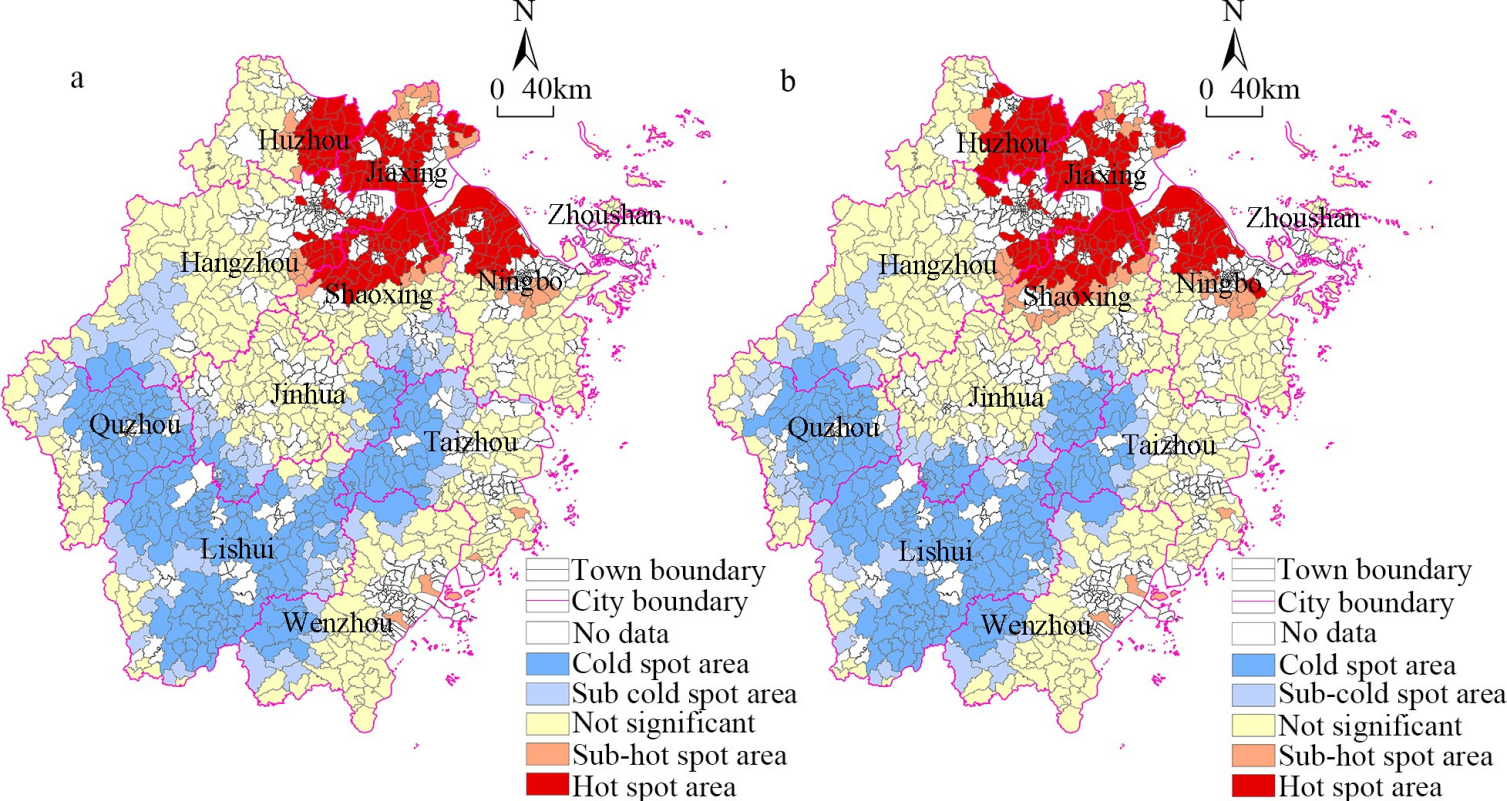
Spatial distribution of cold and hot spots of land economic density in the townships in Zhejiang Province from 2014 to 2019. (a)Year 2014 (b)Year 2019.

The cold–hot spot method was used to visualize the land economic density at the township scale, and the results are shown in Fig 4. From 2014 to 2019, the distribution of land economic density displayed a relatively stable trend, indicating a hierarchical phenomenon primarily constrained to urban units. Cold and hot spots of similar types were predominantly concentrated within individual cities, rather than exhibiting a scattered distribution of multiple types. Specifically, hot spots were primarily concentrated in the northern regions of Zhejiang, while a substantial number of cold spots were distributed across the southern regions of Zhejiang. Specifically, the hot spots were concentrated in the junction between Hangzhou City, Jiaxing City, Shaoxing City, Ningbo City, and Chaozhou City, forming large high-value agglomeration areas. These regions exhibit a high level of economic development and well-established infrastructure, establishing themselves as high-value-added industrial and commercial agglomerations. Driven by favorable resource demands and cost-effectiveness, these areas have attracted significant capital and industrial investments, forming an effective capital-industry-market industrial chain. There were fewer sub-hot spots, and they were distributed around the hot spot areas in Hangzhou City, Shaoxing City, and Ningbo City. The sub-hot spots radiated out from the hot spot areas to promote the integrated development of industry and services and to enhance the comprehensive land use efficiency. The cold-spots were spread over a large contiguous area in the cities of Quzhou, Lishui, and Taizhou. The infrastructure development and level of modernization in these regions are relatively lagging, coupled with their geographical remoteness and constraints imposed by topography. These factors hinder the development of both industrial and service sectors, resulting in a relatively lower land economic density. The sub-cold spots surrounded the cold-spots and were spread over a large area, reflecting the spatial proximity effect of the towns with low land economic densities. In summary, the overall pattern was heterogeneous and did was not effectively improved from 2014 to 2019. The agglomeration effect of the high value areas and low value areas was obvious, reflecting the unbalanced development of the land economies of the towns.

### Comparative analysis of county scale and township scale

**Fig 5:**
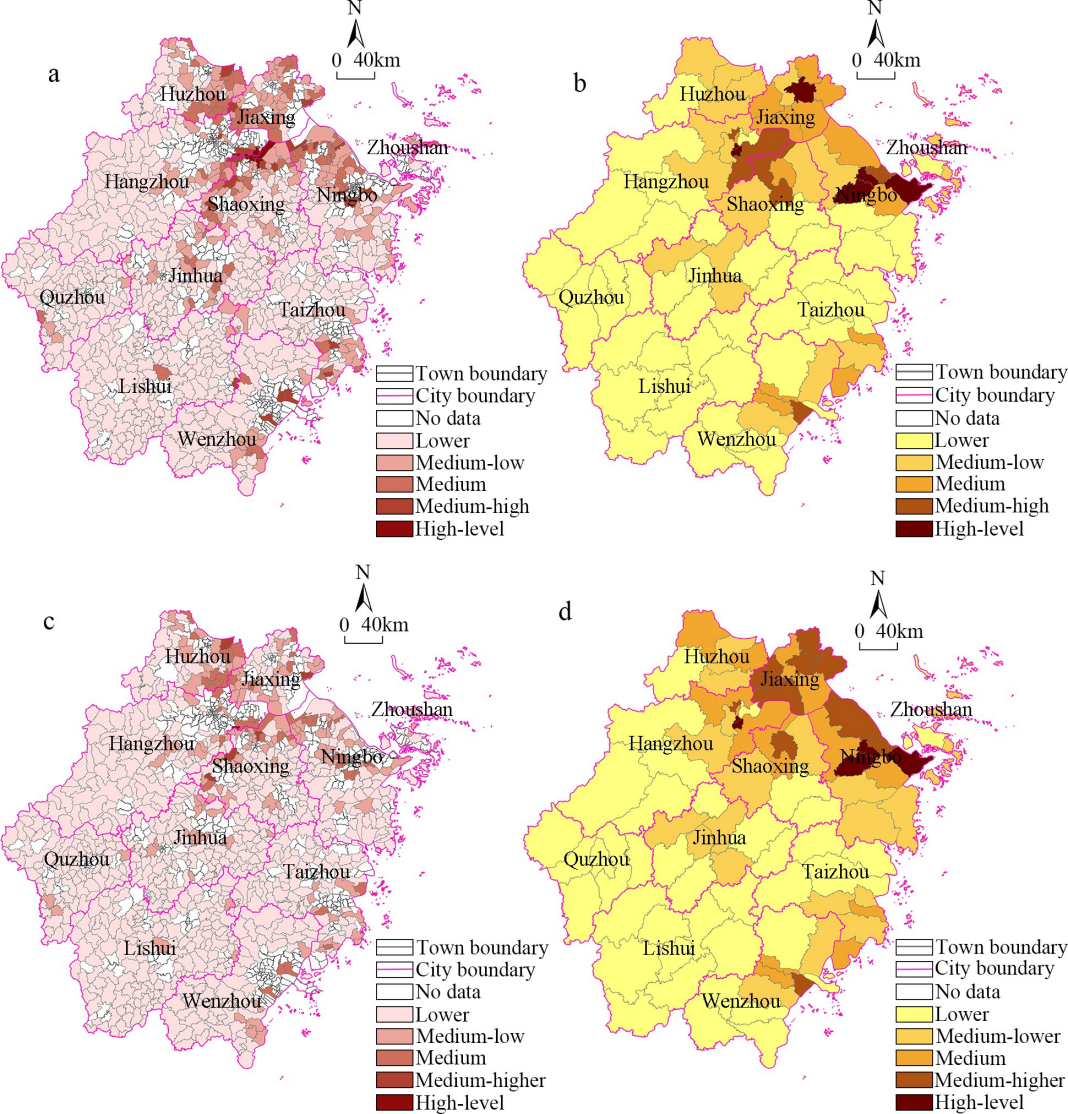
Comparative analysis of the land economic density at the township and county scales. (a)Land economic density of township in 2014 (b) Land economic density of county in 2014 (c) Land economic density of township in 2019 (d) Land economic density of county in 2019.

With the refinement of the scale, the law of economic spatial differentiation is further highlighted, and even the administrative boundaries are broken. Thus, we conducted a comparative analysis of the township scale and the county scale results (Fig 5). Although the overall patterns were similar, the spatial pattern of the township economy was narrower in scope and partially highlights northern Zhejiang. Specifically, the high-level areas at the county scale were not all high-level areas at the town scale, and some of them were transformed into medium-high and medium areas at the township scale, the land economic density in Beilun District and Jiangbei District of Ningbo City is at a high level; however, the township level within the region has not reached the desired level. In central-northern Zhejiang, the high-level areas at the township scale were distributed in a T-sharp along the borders between Hangzhou City, Shaoxing City, Huzhou City, and Jiaxing City. This was different from the medium-high level areas at the county scale, which reflects the phenomenon of breaking the administrative boundaries at the micro-scale. In addition, it should be noted that although most of the areas in southern and western Zhejiang had always been at low levels at the county scale, there were still some high-level areas, such as Fenglin Town and Tong Town. This reflects the positive role of urban characteristics in revitalizing the economy of underdeveloped counties. By fostering distinctive industries or developing specialized services, certain townships can maintain a relatively high level of economic vitality even after refining the scale. In summary, after the scale was refined, the local agglomeration of the economic pattern of the townships was further highlighted, the scope of the high-level areas was significantly narrowed, and the local fragmentation in the undeveloped areas became more obvious.

## Influencing factors

We employ the Geographically Weighted Regression (GWR) model to conduct an in-depth analysis of the factors influencing land economic density. GWR analysis can effectively reveal the spatial non-stationarity of the observed object, which has important application value in disciplines related to geographical locations and has the advantage of being able to directly simulate non-stationary data. The Multi-scale Geographically Weighted Regression (MGWR) and Geographically and Temporally Weighed Regression (GTWR) models serve as extensions of spatial geographically weighted regression models. Although these models are capable of better capturing scale heterogeneity or dynamic features present in the data, the influencing factors addressed in this study do not involve multi-scale or spatiotemporal variations. The primary focus is on overall trends rather than local details. Consequently, GWR is considered a relatively simpler yet effective model choice for this study.

The specific formula is as follows:

$$Y_{i}=\alpha_{0}(S_{i}+T_{i})+\sum_{j=1}^{n}\alpha_{j}(S_{j}+T_{i})x_{ij}+\varepsilon_{i}$$ (4)

where *Y*_*i*_ is the dependent variable of township unit *i*; (*S*_*i*_,*T*_*i*_) is the spatial location coordinates of township unit *i*; and *αj*(*S*_*i*_,*T*_*i*_) is the value of the continuous function *α*_*j*_(*S*_*i*_,*T*_*i*_) in township unit *i*.

Based on previous related studies [1, 6, 20, 31, 32], it was found that most researchers generally selected indicators such as the population, regional area, per capita industrial output, and several industrial structure indexes to analyze the influencing factors (Table 1). Therefore, considering the data availability, completeness, and scientific nature of the indicators, we selected the permanent population, population density, total population, regional area, total number of employed personnel, and proportion of secondary and tertiary personnel to explore the factors influencing the spatial differentiation pattern of the land economic density in Zhejiang Province.

**Table 1:** List of factors influencing the land economic density.

Indicator	Meaning	Reference
Population size	Regional resident population	Liang et al.(2021);Feng et al.(2008)
Industrial structure	The proportion of output value of the secondary and tertiary industries in GDP	Feng et al.(2008);Luo et al.(2010)
Employed population	Total number of employed persons in urban areas	Luo et al.(2010)
Land input	Built-up area	Zhang et al.(2014)
Proportion of output value of tertiary industry	The proportion of output value of the tertiary industry in GDP	Cao(2018)

**Fig 6:**
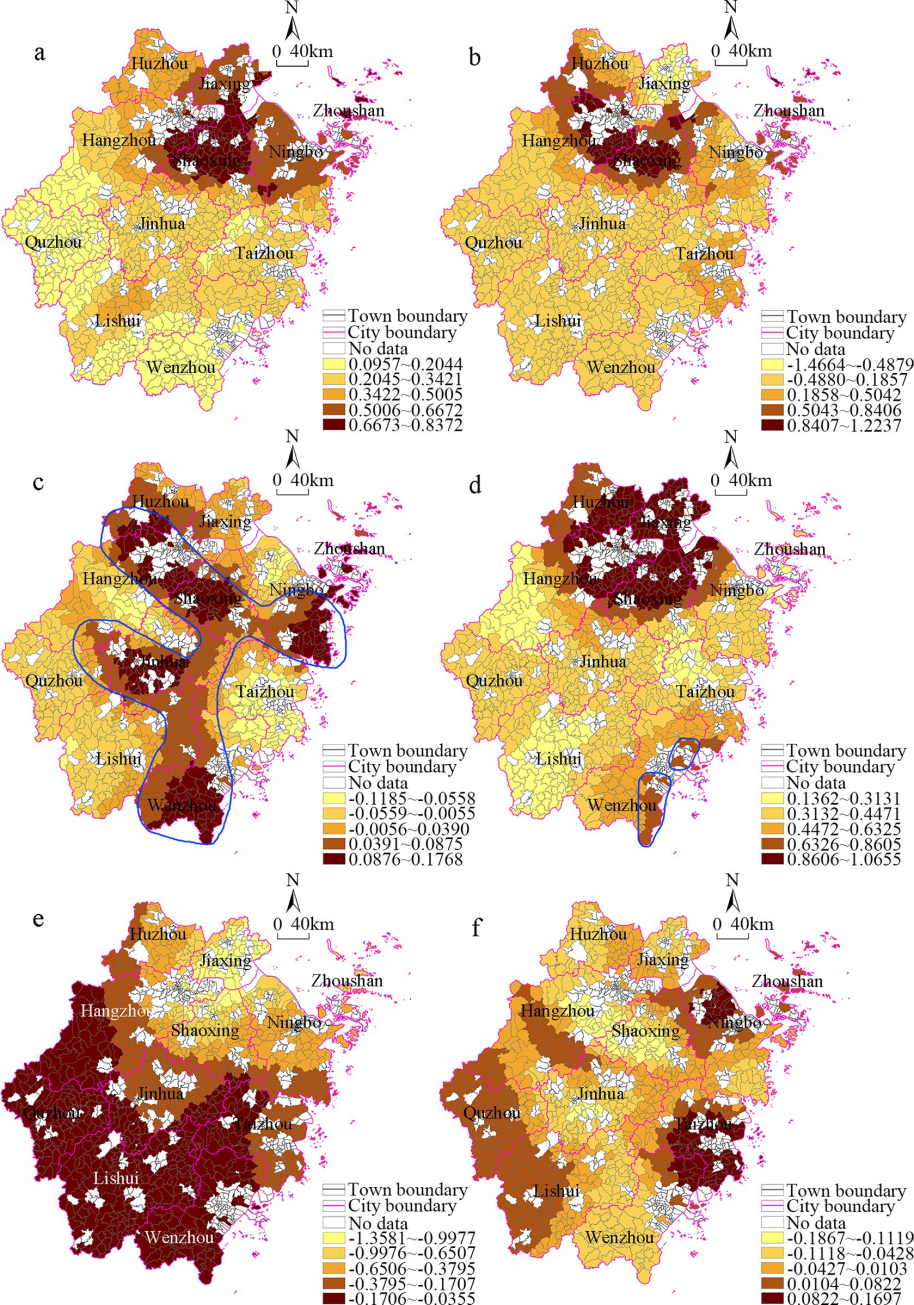
Spatial distribution of regression coefficients of GWR model of land economic density of townships in Zhejiang Province. (a)Total population (b) Population density (c) Total employees (d) Industrial output value per capita (e) Regional area (f) Proportion of secondary and tertiary personnel.

First, the index data were standardized using SPSS software, and then, the stepwise regression model was used for the testing. Since the highest Variance Inflation Factor (VIF) value was 4.82, the variables were not excluded. Second, based on the above results, the data were imported into the ArcGIS 10.2 software and the ordinary least squares regression (OLS) and GWR methods were used to conduct spatial regression analysis. The results revealed that the R2, adjusted R2, and Akaike Information Criterion, corrected (AICc) values of the OLS model were 0.732, 0.729, and 1357.13, respectively. The R2, adjusted R2, and AICc values of the GWR model were 0.879, 0.864, and 789.67, respectively. Thus, fitting effect of the GWR model was better, so we used this model to analyze the influencing factors. Finally, ArcGIS 10.2 was used to visualize the estimated values of the spatial regression coefficients of the six influencing factors, and the results are shown in Fig 6.

According to the absolute values of the regression coefficients, the explanatory powers of the influencing factors were as follows: population density > regional area > industrial output value per capita > total population > proportion of secondary and tertiary personnel > total employees.

We found that the spatial patterns of the resident population and population density obtained using the GWR model were similar to that of the land economic density, especially the distribution of the high-level units in northern Zhejiang. In terms of the population indicators, the state of the population agglomeration reflects the industrialization and urbanization processes, as well as the vitality of the regional economic development, and thus, it provides a basic guarantee and human resource support for regional revitalization. In northern Zhejiang, the commodity economy was developed and the people had a strong sense of innovation, so the value created per unit of land was greater than that in other regions. In turn, the value created from the land was used for improving the urban infrastructure and public service facilities, so the people could enjoy greater benefits, and the land value was further enhanced. In other words, the population agglomeration, land appreciation, and transformation of the industrial structure benignly interacted in the developed regions, which promoted the optimization of the benefits of the population-resources-economy system.

From the spatial pattern of the total employees obtained using the GWR model, the areas with positive regression coefficients exhibited an F-shaped distribution at the connection of the important traffic axis from north to south, and these areas were mainly distributed in the developed counties in central and northern Zhejiang and some of the high economic level counties in Wenzhou City. It was found that these areas not only had a prosperous industrial economy, but they also had a variety of modern service industries. Therefore, by making use of the modern transportation axis, these areas formed a vibrant development belt and economic support belt, which strengthened the regional economic ties and promoted the coordinated development of the urban and rural areas.

The spatial distribution of the per capita industrial output obtained using the GWR model differed from that of the total employees but was similar to the spatial distribution of the land economic density. The consistency of the spatial distributions of the per capita and per land indicators reflects that the high-level areas produced an efficient human-land interaction effect, and it also reflects the coupling effect of the population-industry-economy in space.

Regarding the spatial distribution of the regional area obtained using the GWR model, the regression coefficients were all negative, and the effect was large in the underdeveloped areas with mountainous terrain in southern Zhejiang, reflecting the urgent need to further change the location conditions in these areas. Based on the status quo of the regional economic development, although these areas had some resource advantages and the economic strength of the townships did gradually improve, owing to the limitations imposed by the terrain, the influence of the urban areas in the counties on the surrounding rural areas was not strong, and the driving effect of the characteristic townships on their adjacent areas was not obvious. However, the spatial pattern of the proportion of secondary and tertiary personnel obtained using the GWR model was completely different from the distribution in the regional area, which also indicates that the level of the modern service industry in the southern Zhejiang was not high. The areas with positive high regression coefficients were mainly distributed in Ningbo City and at the junction between the northeastern part of Wenzhou City and the urban area of Taizhou City, which reflects the high level of industrialization and urbanization, as well as some coastal openness in these regions.

## Discussion

The main purpose of analyzing the spatial distribution of the land economic density at the township scale and its influencing factors was to understand the inner spatial mechanisms of the underlying economy, to provide support for urban system planning, and to improve the resource balanced allocation of the population, land, and industry. However, as was mentioned in the literature review, most studies drew relevant conclusions based on measurement indexes of per capita indicator data, especially the per capita GDP, and they rarely comparatively analyzed the benefits of the land outputs. Therefore, when analyzing regional economic differences, previous researchers ignored the spatial correlation effects of the geographical location and land resources and the boundary effects in micro-scale studies. For example, when analyzing the regional economic disparity in Zhejiang Province, some researchers focused on the spatial pattern explained by aggregate indicators and ignored the spatial effects of the population-land-industry system based on the land economic density index. For specific cases, the Wenzhou model proposed by previous scholars is not only based on advanced ideas, transformation of the industrial structure, and talent innovation activities, but it is based on the coupled and coordinated mechanism of the population, land, and industry. In this study, the factors influencing the land economic density in Wenzhou City were analyzed in terms of the economic input-output efficiency and the economic output per capita, and the human-land effect of the local high-output townships was confirmed.

Influenced by factors with multiple dimensions such as the socio-economic foundation, human-land relationship, urban-rural structure, and township spatial management, it is impractical to rely solely on the single indicator (i.e., the industrial economic density) to analyze the economic pattern at the township scale. Therefore, in this study, the analysis of the influencing factors such as the population density, total employees, proportion of secondary and tertiary personnel, and industrial output value per capita were strengthened to comprehensively investigate the influencing factors. If we unilaterally emphasize the measurement results of a certain indicator, it may lead to insufficient research breadth and depth, and it becomes difficult to link the index to the actual development status. In previous studies, evaluations of the regional economy at the township scale focused on the output value of the industrial enterprises and the formation mechanism of special industrial clusters, while the supportive influence of the land was ignored. The improvement of the total economic volume was the focus, and the land carrying capacity and land planning of the township were neglected, resulting in the unreasonable use of land resources and the disorderly expansion of township land construction. Furthermore, the results of these studies may lead to a situation where the actual economic development runs counter to the planning assumptions of the town economy. Therefore, it is important to analyze the spatial pattern using industrial economic density indicators and to strengthen the comprehensive analysis of the population, land, and industry, as well as their multi-scale correlation effects.

It should be noted that in this study, a comparative analysis of the land economic density was conducted at multiple scales, and interactive analysis of the population-land-industry system was integrated into the evaluation results. Thus, our results are more conducive to understanding the evolution of the industrial economic pattern in Zhejiang Province. Owing to the difficulties in obtaining data and other reasons, most previous studies chose a single year for township economic analysis, which leads to limitations and makes it difficult to provide stability from a comparative perspective. In this study, two years were chosen to analyze the spatial pattern of the industrial economy, which improved our understanding of the spatial evolution. The results of the analysis revealed that the growth center in the north-central region of Zhejiang Province shifted to the west. In reality, the north-central region relies on the industrial base and attaches importance to e-commerce, as well as characteristic townships and beautiful villages, keeping these areas at a high level. Driven by Taobao Village and the live-streaming market economy, local industrial enterprises such as packaging and design, logistics enterprises, and consulting services have rapidly emerged and have formed an interdependent e-commerce industry chain, promoting the shift in the center of the regional economy to the west. In addition, the local government has made plans based on enterprise agglomeration, resource endowment, and e-commerce culture, forming an efficient link between the industry and land, a combination of theme parks and livable ecology, and the integration of shopping consumption and tourism. In addition, the development of the industrial economy can quickly promote the improvement of the infrastructure, the prosperity of industry, and the ecological livability. In turn, the implementation of the beautiful countryside policy has optimized the land layout of the townships and promoted upgrading of industry, which has led to a link between agriculture, industry, and services and has further enhanced the comprehensive use of the land.

## Conclusions

Based on the land economic density index, in this study, the correlation coefficient, spatial interpolation analysis, cold–hot spot analysis, and geographical weighted regression methods were used to explore the differences in the spatial distribution of the industrial economy in Zhejiang Province at the township scale, and its influencing factors were analyzed. Based on the spatial distribution of the CV values, the high variance areas were distributed in the northeastern part of Lishui City; the medium variance areas with CV value were distributed in central-northern Zhejiang and its southern outskirts; and the low variance areaswere mainly distributed in northern Zhejiang, which is similar to the distribution pattern of the high-level economic areas at the county scale. Based on the spatial interpolation analysis, the distribution of the high-level agglomeration areas was characterized by one core area and two sub-core areas. The main core area was located at the junction between Hangzhou City, Shaoxing City, and Jiaxing City, and the two sub-core areas were located in Yuyao City and the main urban area of Ningbo City. In addition, several small-scale agglomeration areas composed of medium and high-level units were distributed in Wenzhou City. There were high-value agglomerations and low-value agglomerations in the spatial correlation patterns obtained using the spatial auto-correlation method, areas with similar characteristics, whether hotspots or coldspots, tend to concentrate within a single city, rather than displaying a scattered distribution across multiple types. The hot spots and sub-hot spots were distributed in northern Zhejiang; and the cold spots formed a large-scale agglomeration in Quzhou City, Lishui City, Taizhou City, and several other cities in southern Zhejiang. Compared with the county scale results, the spatial scope of the high-level area in northern Zhejiang was significantly smaller at the township scale, and the high-level agglomeration areas along the southeast coast changed into a cluster of several townships. Based on the absolute value of the regression coefficient, the order of importance of the influencing factors was as follows: population density > regional area > industrial output value per capita > total population > proportion of secondary and tertiary personnel > total employees.

In this study, a variety of spatial analysis methods were used to analyze the spatial variation pattern of the township economy in Zhejiang Province at the township scale and to reveal the mechanism through influencing factor analysis using the GWR model to explore the microeconomic laws. However, due to the problems of data acquisition and the consistency of the indicators, the land density of the township economy was only analyzed in 2014 and 2019, so the long-term dynamic changes could not be analyzed. Thus, the comparative analysis of multiple time periods can be strengthened in the future. In addition, although we conducted a more micro-scale analysis, we did not analyze the actual problems of the specific townships and the optimization of the spatial pattern. Therefore, in future research, we should strengthen the analysis of the specific problems such as improvement of the land efficiency, adjustments to the industrial structure, infrastructure improvements, and other development issues in specific townships, and targeted paths and spatial planning schemes should be proposed. It should be noted that the economic levels of the townships in southern Zhejiang were not high, and they still face problems such as poor transportation to an inconvenient location and a weak economic foundation. In the future, we should strengthen the research on the mechanisms of land-use, population agglomeration, and industrial transformation in southern Zhejiang based on in-depth local investigations to provide support for rural revitalization.

## Supporting information

S1 Data (XLS)
